# Total body irradiation (TBI) combining volumetric modulated arc therapy (VMAT) for thorax at standard source axis distance (SAD) with intensity modulated radiation therapy (IMRT) at extended SAD

**DOI:** 10.1002/acm2.70529

**Published:** 2026-03-18

**Authors:** Marc Delaperrière, Markus Hirt, Christian Felix Schulz, Frank André Siebert

**Affiliations:** ^1^ Department of Radiation Oncology University Hospital Schleswig‐Holstein (Universitätsklinikum Schleswig‐Holstein, UKSH) Kiel Germany

**Keywords:** bilateral TBI, eSAD, hybrid TBI, IMRT TBI, TBI, total body irradiation

## Abstract

**Purpose:**

Total body irradiation (TBI) is an important part of conditioning regimens prior to hematopoietic stem cell transplantation (HSCT). At our institution, a conventional TBI technique employing bilateral fields at extended source‐to‐axis distance (eSAD) and a solid three‐dimensional (3D) compensator has been standard practice since the 80s. However, its ability to minimize lung dose remains limited.

**Aims:**

This study aims to modernize the existing TBI technique using the capabilities of our treatment planning system (TPS). The proposed hybrid method replaces solid compensators with virtual compensation by combining bilateral intensity‐modulated radiotherapy (IMRT) fields at eSAD of 500 cm with volumetric modulated arc therapy (VMAT) fields at standard SAD 100 cm for the thoracic region.

**Methods:**

At first, the Anisotropic Analytical Algorithm (AAA) V16 and AcurosXB (AXB) V16 dose calculation algorithms of the Eclipse TPS (Varian) were commissioned for use at eSAD. Dosimetric measurements were performed in water, solid water, and inhomogeneous phantoms. Subsequently, a treatment planning strategy was developed to minimize the influence of positioning uncertainties on lung dose. Anonymized computed tomography (CT) datasets representing diverse patient anatomies were utilized for treatment planning evaluation. Final validation was conducted through an end‐to‐end test using an anthropomorphic phantom. SAD VMAT and eSAD IMRT fields were verified using a two‐dimensional (2D) dosimetry chamber array positioned at both caudal and cranial thoracic levels under SAD and eSAD setups. The resulting cumulative dose was then compared with the AXB‐calculated dose.

**Results:**

At eSAD, mean measured–calculated dose deviations were < 2% for AXB and < 4% for AAA, with confidence limits of 3.5% and 5%, respectively. These results affirmed the clinical viability of both algorithms, with AXB providing superior dose calculation accuracy. Planning studies showed consistent PTV coverage with lung dose reduction across diverse anatomies, and end‐to‐end phantom validation confirmed the workflow practicability. Verification of summed dose using the 2D array, with 95% of evaluated points meeting a gamma criterion of 5 mm and 5%, provided additional confidence in the dosimetric robustness of the treatment concept.

**Conclusion:**

This study showed the feasibility of a novel hybrid eSAD IMRT/VMAT TBI technique providing a clinically viable approach enabling effective lung dose sparing while maintaining the robustness of large‐field irradiation.

## 1 INTRODUCTION

Total body irradiation (TBI) is a form of external beam radiotherapy frequently combined with chemotherapy as part of the conditioning regimen prior to hematopoietic stem cell transplantation (HSCT) and is employed in the treatment of some hematologic malignancies and certain genetic disorders. TBI delivers a high dose of radiation to the entire body with the dual aim of eradicating residual malignant cells and inducing immunosuppression to facilitate engraftment.^1^, ^2^ However, TBI is associated with potential toxicities, among which pulmonary toxicity represents a clear clinical concern^3^, ^4^

Various non‐standardized prescription dose (PD) and fractionation regimens are used for TBI^2^, ^5^ depending on the disease, the patient's age and condition, the chemotherapy regimen and the type of transplant, allogeneic or autologous. In our clinic, typical regimens include PD 2 Gy in a single fraction, PD 8 Gy in 4 fractions over 2 days and PD 12 Gy in 6 fractions over 3 days with a prescribed lung dose reduction down to 10 Gy mean.

To minimize the risk of pulmonary toxicity and mitigate the potential lethality associated with high‐dose irradiation, TBI planning and treatment delivery must be carried out with accuracy and robustness.

Irradiation techniques, also non‐standardized, are employed for TBI including extended source‐to‐axis distance (eSAD) large‐field setups, field patching, and dynamic techniques^1^, ^6^, ^7^ as well as more recent techniques such as volumetric modulated arc therapy (VMAT)^1^, ^6^, ^7^, ^8^, ^9^, ^10^ and helical tomotherapy.^11^, ^12^ The choice of a technique is influenced by a number of factors, including the available radiotherapy equipment, the geometric constraints of the treatment room, and past clinical experience and technical expertise acquired in the facility.

**FIGURE 1:**
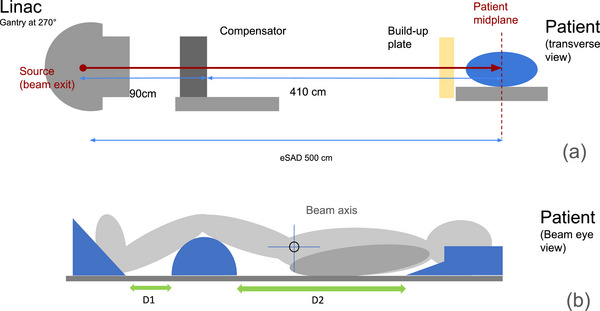
Setup for patient irradiation with solid compensator at extended source‐axis‐distance (eSAD) of 500 cm (a) overall view, (b) patient setup with immobilization devices.

The TBI technique used in our clinic, which was originally developed in 1983 and has undergone some modifications since its creation,^13^ uses a bilateral field approach, with the patient positioned on a wall‐mounted treatment couch at an eSAD of 500 cm (Figure 1a). The patient is immobilized with cushions placed at reproducible positions beneath the head, knees, and feet, arms kept close to the body (Figure 1b). This setup enables a 200 × 200 cm^2^ open field at eSAD 500 cm, which corresponds to a 40 × 40 cm^2^ open field at the isocenter of a conventional linear accelerator (linac). The linac C‐arm (gantry) is rotated at 270°, and the patient is first irradiated in the supine position on the right lateral side with a 15 MV photon field. The patient is then repositioned to receive irradiation on the left lateral side, also in supine position, with a second 15 MV photon field. For each lateral exposure, a manufactured solid tin‐paraffin compensator is placed in the beam path on the standard treatment couch, 90 cm from the source, to achieve dose homogeneity and, in PD 12 Gy treatments, reduce the dose to the lungs. In addition, a 19 mm thick plate of polymethyl methacrylate (PMMA), so‐called build‐up plate, is placed in the beam close to the patient to increase the surface dose.

The 3D shapes of the solid compensators are calculated using a projection algorithm applied to the patient's whole‐body CT scan, acquired in the same immobilization position as later for treatment, considering radiological length differences in the body. The compensators are then fabricated by milling 8 cm thick polystyrene blocks, which are then filled with a tin‐paraffin mixture, an approach that necessitated logistical and manufacturing efforts. Linac monitor units (MUs)—1 MU adjusted to 1 cGy at percentage depth dose (PDD) maximum in reference conditions (according to German norm DIN 6800)—are calculated to deliver 1 Gy per lateral treatment field (for a total of 2 Gy per fraction) at the intersection of the beam's central axis and the patient median plane. Dose calculations were based on tissue‐phantom ratio (TPR) data acquired in a water phantom at eSAD 500 cm, combined with a basic model of exponential dose attenuation through the compensator material.

Patient positioning is verified using 4 computed radiographs of max. size 43.0 × 35.4 cm (Fuji Computed Radiography, FCR, Tokyo, Japan) acquired with cassettes placed behind the patient during delivery of the initial 150 MUs of the first treatment fraction.

While point dose calculations based on measured data tables is a robust and reliable approach to minimize gross dosimetric errors, it also introduces uncertainties: factors such as variations in scattered dose contributions, spectral beam hardening in the compensator and the off‐axis dependence of linear attenuation^14^ are not considered. Furthermore, the use of two lateral beams limits the ability to reduce the dose to the lungs, as any reduction in the pulmonary exposure concomitantly reduces the dose to the patient's mediastinum and thoracic cage. This limitation is a drawback compared to alternative approaches like AP‐PA fields or newer multi‐isocentric VMAT techniques, which offer improved organ‐at‐risk sparing.^15^

**FIGURE 2:**
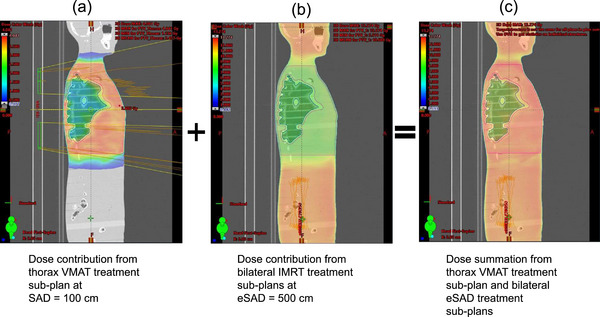
Combining a VMAT thoracic treatment sub‐plan at SAD 100 cm with a bilateral IMRT treatment sub‐plan at eSAD 500 cm.

In this study, we therefore propose a novel TBI approach that eliminates the need for a physical compensator by integrating intensity‐modulated radiation therapy (IMRT) treatment sub‐plans delivered at eSAD with an optional VMAT treatment sub‐plan targeting the thoracic region at a standard source to axis distance 100 cm (SAD) (Figure 2).

**FIGURE 3:**
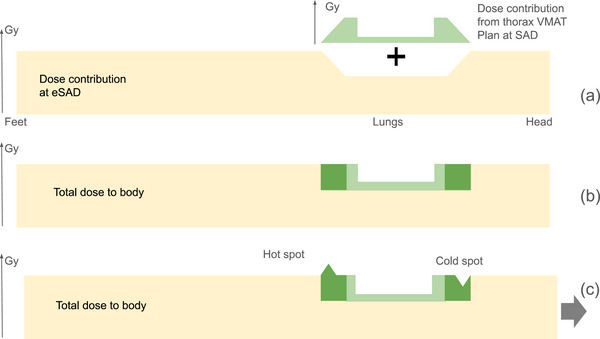
Dose summation strategy combining the thoracic VMAT treatment sub‐plan and eSAD IMRT fields. (a) Lung dose reduction is ideally achieved exclusively through the VMAT treatment sub‐plan. (b) Ideal summation of the thoracic VMAT and eSAD IMRT dose distributions. (c) A positional shift might result in dose hot and cold spots but does not affect the lung dose.

To ensure that the cumulative dose from the different sub‐plans matches the prescription without creating hot or cold spots, particularly in the lungs, three principles form the core of the novel approach schematized in Figure 3. First, reducing lung dose relative to the mediastinum and thoracic cage in the VMAT treatment sub‐plan only, while maintaining a uniform dose to the whole thorax in the eSAD treatment sub‐plans. Second, creating a region at the edges of the thoracic volume to control and reduce dose gradients in both SAD and eSAD treatment sub‐plans and third, accounting for differences in beam divergence and isocenter positions between the SAD and eSAD treatment sub‐plans. The last two principles are known from multi‐isocenter studies.^6^, ^16^

Hybrid strategies for TBI planning, mixing two techniques, have been described earlier, like the Stanford technique, mixing VMAT and APPA fields^8^, ^17^ or notably the two compound methods presented by Hansen et al. called exVMAT and exIMRT.^18^ The implementation presented here is distinct and augments this category of techniques. Building on the clinic's prior bilateral field TBI experience, it preserves the eSAD 500 cm setup, patient positioning and immobilization, 10 Gy lung dose reduction objective and strengthens the existing quality framework.

## 2 MATERIALS AND METHODS

Implementation of a TBI treatment hybrid technique using our treatment planning system (TPS), (Eclipse V16, Varian Medical Systems, Palo Alto, CA) firstly necessitated comprehensive commissioning of the dose calculation algorithms at eSAD for our Varian TrueBeam linac equipped with a Varian Millennium multi leaf collimator (MLC). Additionally, the clinical workflow from dose prescription to treatment delivery and reporting required revision, including definition of dedicated quality assurance (QA) procedures.^1^, ^19^ These updates were formalized in a new standard operating procedure (SOP).

The following steps were therefore defined for validation of the TBI hybrid technique:

1. Commissioning of TPS's algorithms at eSAD
2. Define and validate a standardized treatment planning protocol and workflow
3. End‐to‐end test using an anthropomorphic phantom

### 2.1 Commissioning of TPS's algorithms at eSAD

**FIGURE 4:**
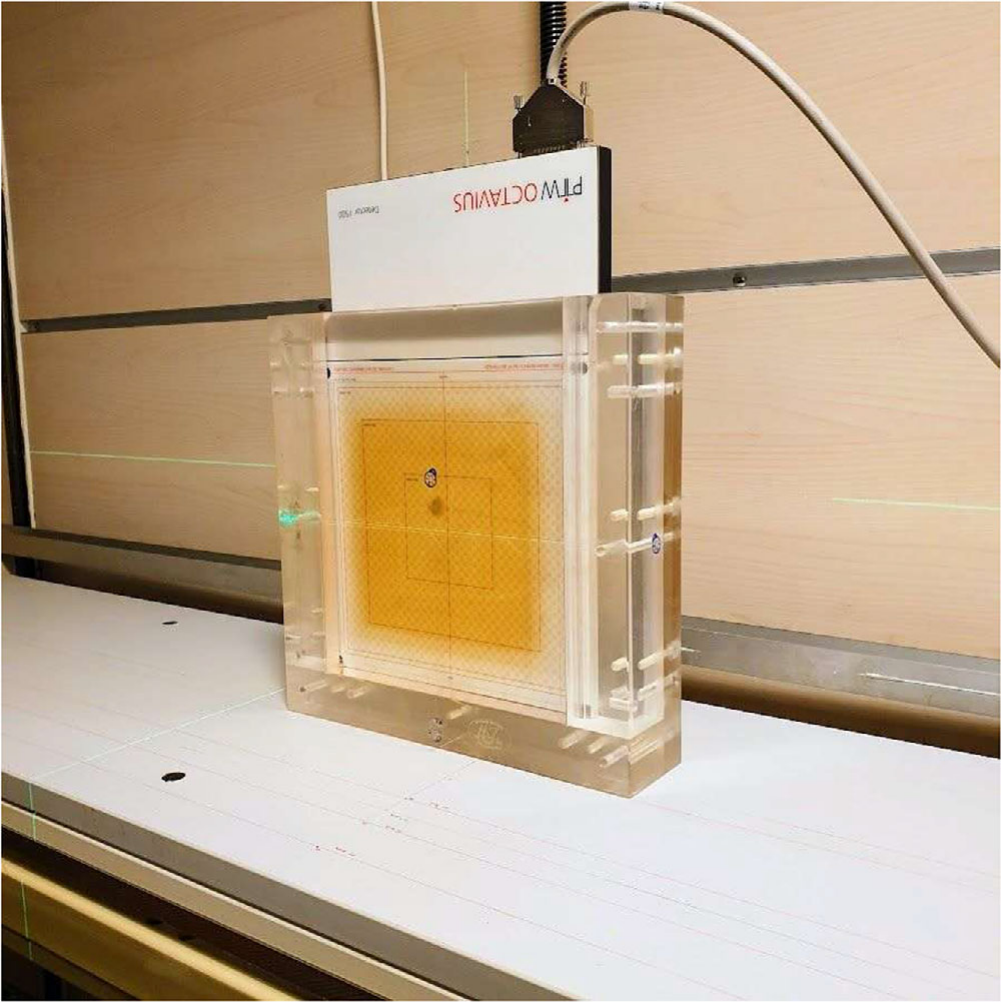
PTW Octavius 1500 2D array embedded in a 40x40x10 cm^3^ PMMA phantom positioned on the TBI wall mounted couch.

To assess the accuracy of the Varian Eclipse TPS algorithms, Analytic Anisotropic Algorithm (AAA) v16 and Acuros XBs (AXB) v16 under eSAD conditions, a series of dosimetric measurements were performed using a calibrated Farmer‐type ionization chamber (TM30013, PTW, Freiburg, Germany) connected to a PTW Unidos electrometer. Measurements were conducted in accordance with the recommendations of AAPM TG‐17 (Dyk et al.) and the German standard report DIN 6873‐1. Absolute dose measurements adhered to the dosimetric protocol specified in DIN 6800–2. All commissioning measurements were performed for a 15 MV photon beam with build‐up plate, as intended to be clinically applied in TBI at eSAD. They included point measurements in a 30×30×30 cm^3^ water phantom for horizontal beam setups (PTW 41023) for 1.a) output factors & depth doses, 1.b) dose profiles at 3 depths, 1.c) MLC sliding windows with width of 2 to 20 mm. 1d) Sample 3D and MLC‐modulated fields were also measured in a thoracic inhomogeneous phantom with lung and bone‐equivalent materials (CIRS, Sun Nuclear, Melbourne, FL) and 1.e) Beam profiles for a 4 × 4 cm^2^ field and half profiles for larger fields (4 × 10 to 4 × 40 cm^2^) measured using a cross‐calibrated 2D array of dosimetry chambers (Octavius 1500, PTW) embedded in a PMMA phantom (40 × 40 × 10 cm^3^; Figure 4). For large fields the phantom was positioned vertically at 20 cm intervals along the TBI couch, with the chamber array placed at 5 cm depth in the PMMA and an eSAD of 500 cm. The field width was limited to 4 cm in one direction to avoid irradiation of the 2D array electronics. Corresponding dose distributions were computed in the TPS using both AAA and AXB algorithms (transport mode set to dose‐to‐medium and reporting mode set to dose‐to‐water) and using a structure model of 19 mm thickness and 160 HU to simulate the PMMA build‐up plate in front of the phantoms located at extended source surface distance (eSSD) 470 cm.

Algorithm accuracy was assessed for measurements (1.a–1.d) following the methodology cited by the IAEA,^20^ incorporating average dose deviations (AD) and the confidence limit (CL) approach as defined by Welleweerd and Venselaar^21^ Equation 1. PTW array profile data (1.e) were analyzed with PTW Verisoft V8.1 for the 4 × 4 cm^2^ field, while larger‐field measured profiles were merged and compared to calculated fields in a datasheet (Microsoft Excel 365, USA).

(1) $$CL=\left|average\;deviation\right|+1,5\times \left(standard\;deviation\right)$$

### 2.2 Define and validate a standardized treatment planning protocol and workflow

**FIGURE 5:**
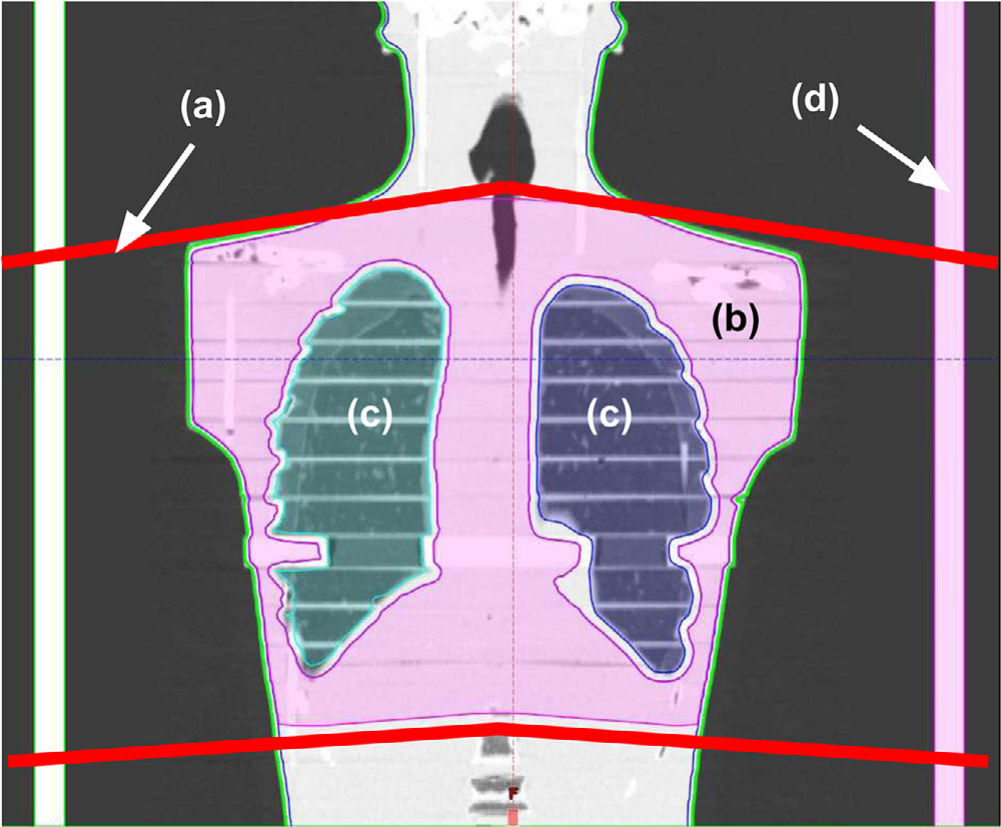
Accounting for (a) beam divergence for the (b) PTV Thorax delineation. (c) Lung contours. (d) Build‐up plate model.

At first, the planning target volume (PTV) is delineated in the TPS contouring workspace by applying a 3 mm isotropic inner margin to the body contour and subtracting the lung contour expanded by a 5 mm margin. A “thorax PTV” is also defined by cropping the PTV 2 cm cranial to 2 cm caudal from lungs accounting for beam divergence at eSAD for later field superposition (Figure 5). An additional 3 cm cranial and caudal volume is also delineated to accommodate for controlled dose gradients. The set planning objectives for the PTV relative to PD were derived from Hoeben et al.^5^ who defined a rationale for VMAT and helical Tomotherapy TBI: D98% ≥ 85%, V95% ≥ 90%, V110% < 3‐5%, V120% < 1% and a mean lung dose ≤ prescribed lung dose.

The treatment plan is then divided into treatment sub‐plans as provided in the overview Table 1.

**TABLE 1:** Overview of the treatment sub‐plans used in the hybrid SAD VMAT and eSAD IMRT technique.

Treatment sub‐plan	Treatment fields	Patient set‐up	Comment
“SAD VMAT thorax”	2 to 4 volumetric modulated arcs, Photon Energy 6 MV	HFS, on standard treatment couch	Only if a lung dose reduction is required
“eSAD patient right”	IMRT fields at gantry 270°, collimator 5° (“R IMRT 1”) and 350° (“R IMRT 2”), Photon Energy 15MV	HFS, on dedicated TBI wall mounted couch	“SAD VMAT thorax” used as base plan & build up plate model added to patient CT
“eSAD patient left”	IMRT fields at gantry 270° collimator 355° (“L IMRT 1”) and 10° (“L IMRT 2”), Photon Energy 15MV	FFS, on dedicated TBI wall mounted couch	“SAD VMAT thorax” used as base plan & build up plate model added to patient CT

The VMAT thorax treatment sub‐plan, is calculated first in the TPS using 6 MV photon beams at a dose rate conservatively reduced to 40 MU/min, consistent with established rapid arc TBI techniques^9^, ^22^ and close to the dose rate range at eSAD used with the prior solid compensator technique. The treatment isocenter is chosen centered mid of the PTV thorax. The VMAT treatment sub‐plan is optimized to deliver about 30% of the total prescribed dose (4 Gy for a PD 12 Gy) to the thorax PTV with the objective of already achieving a 2 Gy dose reduction in the lungs.

The eSAD lateral treatment sub‐plans use 270° 15 MV photon fields, operating at a nominal dose rate of 600 cGy/min at isocenter, which corresponds to 24 cGy/min at eSAD 500 cm. Each treatment sub‐plan, one for the patient right side (“eSAD patient right”, patient in head first supine (HFS) orientation) and one for the patient left side (“eSAD patient left”, patient in feet first supine (FFS) position) include two IMRT fields optimized to ensure a uniform dose distribution to the PTV, accounting for any dose already delivered by the VMAT thorax treatment sub‐plan (so called in the optimization process “base plan”). Creating eSAD treatment sub‐plans involved intermediate steps such as adding the build‐up plate model to the whole‐body patient structure set and initially optimizing parameters using the whole‐body CT in HFS orientation only, with gantry angles of 270° and 90°.

The treatment planning method was tested using anonymized CT datasets from three representative patients: an adult male (180 cm), an adult female (160 cm), and a pediatric patient (110 cm). Two different dose prescriptions were evaluated: a PTV PD of 12 Gy in 6 fractions with a reduced lung dose reduction to 10 Gy (thorax and eSAD treatment sub‐plans are then necessary), and a PTV PD of 8 Gy without lung dose reduction (eSAD treatment sub‐plans only are required). Treatment plans were optimized to ensure homogeneous PTV coverage while aiming to keep total beam time under 30 min. The goal of this test was to assess the applicability of the treatment planning method across different patient morphologies and confirm that planning objectives can be reached.

The treatment planning steps described previously constitute the core of the SOP within the clinical workflow. Planning CT imaging protocol, patient immobilization, and position verification were only minimally modified from the clinic's established procedures with solid compensation. Nevertheless, additional quality assurance measures inherited from conventional IMRT and VMAT treatment protocols^23^ were added:

1. Portal Dosimetry: beam fluences are verified for the VMAT treatment sub plan as well as for individual eSAD IMRT fields using the linac integrated electronic portal imaging device (EPID) at an SSD of 100 cm. A gamma criterion of 3 mm and 3% dose difference between measured and planned dose maps is applied for evaluation.
2. Phantom based verification: IMRT fields are further validated using an RW3 solid water phantom positioned on the TBI couch at eSAD. Measurements are acquired with a Farmer dosimetry chamber connected to an electrometer, both on the central beam axis, representative for abdominal dose and at an off‐axis location corresponding to the mid‐lung location.
3. EPID cone beam patient positioning verification before delivering the VMAT treatment sub‐plan to the patient.

Finally, a checklist for peer review, including defined acceptance criteria for plan approval was incorporated into the SOP.

### 2.3 End‐to‐end test using an anthropomorphic phantom

**FIGURE 6:**
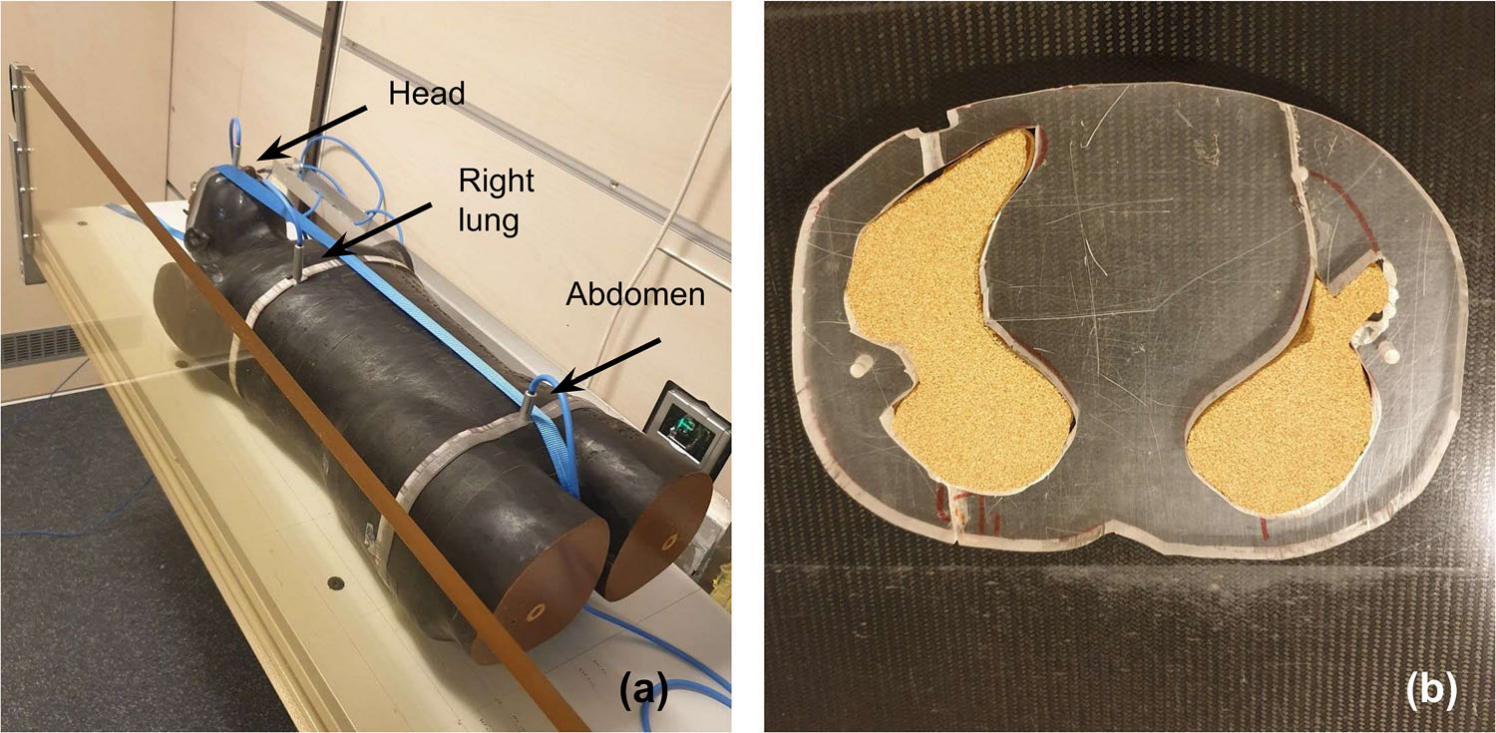
Farmer chamber positions (a) in head, lung and abdomen in the Alderson phantom with PMMA inserts. (b) PMMA insert at lung height with cork to simulate lung tissue.

The treatment workflow, from planning CT imaging through to dose delivery was evaluated end‐to‐end using an anthropomorphic Alderson phantom, modified with PMMA inserts, for Farmer chamber placement, one of them containing cork to simulate lung tissue (Figure 6). Anthropomorphic phantoms are widely used for validation of TBI treatments^18^, ^24^, ^25^ as they partly replicate human morphology, enabling clinically relevant treatment simulations.

A treatment plan was generated using the dataset of the modified Alderson phantom for PD 12 Gy to the PTV with a reduced lung dose of 10 Gy using MLC sliding window motion. In order to test our patient specific QA protocol, plan verification followed our SOP, using the EPID to check the VMAT treatment sub‐plan and dosimetry chamber measurements in the RW3 phantom for the IMRT eSAD treatment sub‐plans.

However, as part of end‐to‐end testing, additional verification of the cumulative dose impact in the thoracic region from combined SAD and eSAD delivery setups was performed: Sub‐treatment plans were delivered to a 2D array detector embedded in the previously described PMMA phantom (Figure 4). Measurements were acquired at two middline thoracic positions (cranial and caudal and at an additional caudal position displaced 8 cm laterally corresponding to the upper right lung, under both SAD and eSAD conditions. For each treatment sub‐plan, measured dose profiles were compared with AXB calculations using a 5 mm and 5% dose gamma criterion, increased relative to standard tolerances to account for the extended eSAD distance, associated geometric uncertainties, and the use of large field sizes. Measured doses were summed using an in‐house MATLAB script (V2023b, MathWorks, USA) and compared with the calculated AXB dose sum using Verisoft.

**FIGURE 7:**
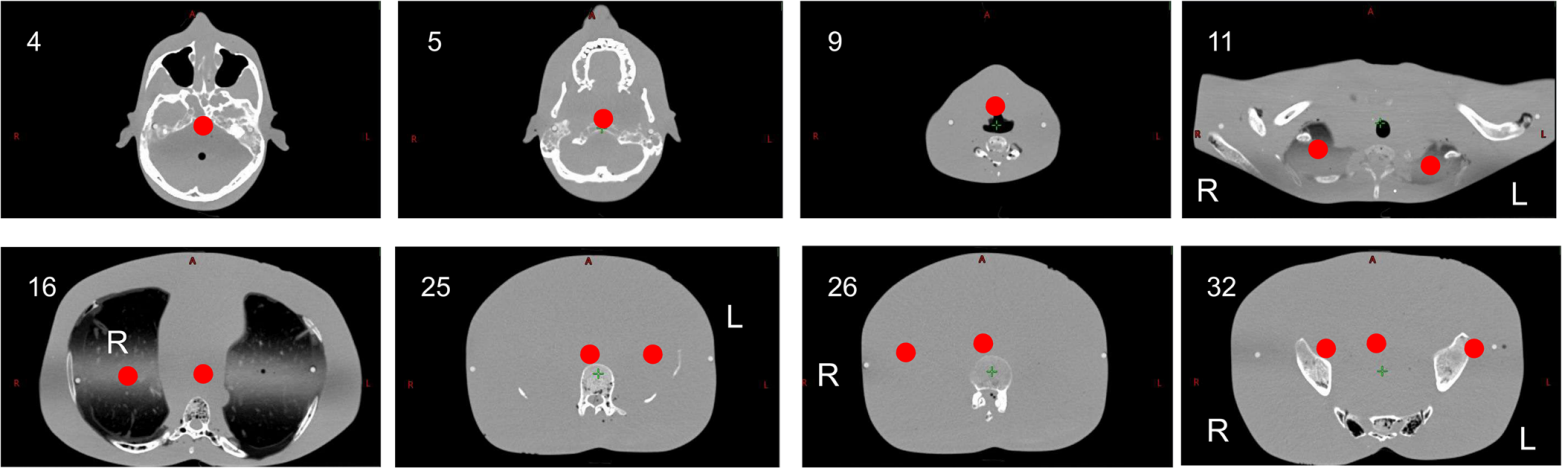
Positions of the 12 TLDs are marked with a red dot in the CT images of the Alderson phantom.

The Alderson phantom was then set‐up at both SAD and eSAD for delivery of one treatment fraction after position verification (with cone Beam CT images for the SAD sub‐treatment plan and with FCR for the eSAD sub‐treatment plans). Farmer chamber dose measurement at locations of head, right lung and abdomen were performed (Measurements 3.a.). Pre‐calibrated thermoluminescence dosimeters (TLD‐100) were placed additionally at different locations in the phantom (Figure 7 (Measurements 3.b.)). These TLDs were picked from a greater batch for deviations of less than 2% over 3 verification measurements for 2 Gy in the 30 × 30 × 30 cm^3^ water phantom and calibrated to the mean of the two last measurements. Finally, to assess the dosimetric robustness of the treatment plan, that is, tolerance to position errors, ± 1 cm offset was applied in the TPS to the phantom's longitudinal and sagittal positions in the eSAD treatment sub‐plans, while no offsets were applied in the VMAT treatment sub‐plan. Acceptance criteria were based on those proposed by Seravalli E et al.^6^ who evaluated robustness of VMAT TBI with multiple isocenter: V90% PD > 95%, V110% PD < 3% and V120% PD < 1%.

## 3 RESULTS

### 3.1 Commissioning of TPS's algorithms at eSAD

**FIGURE 8:**
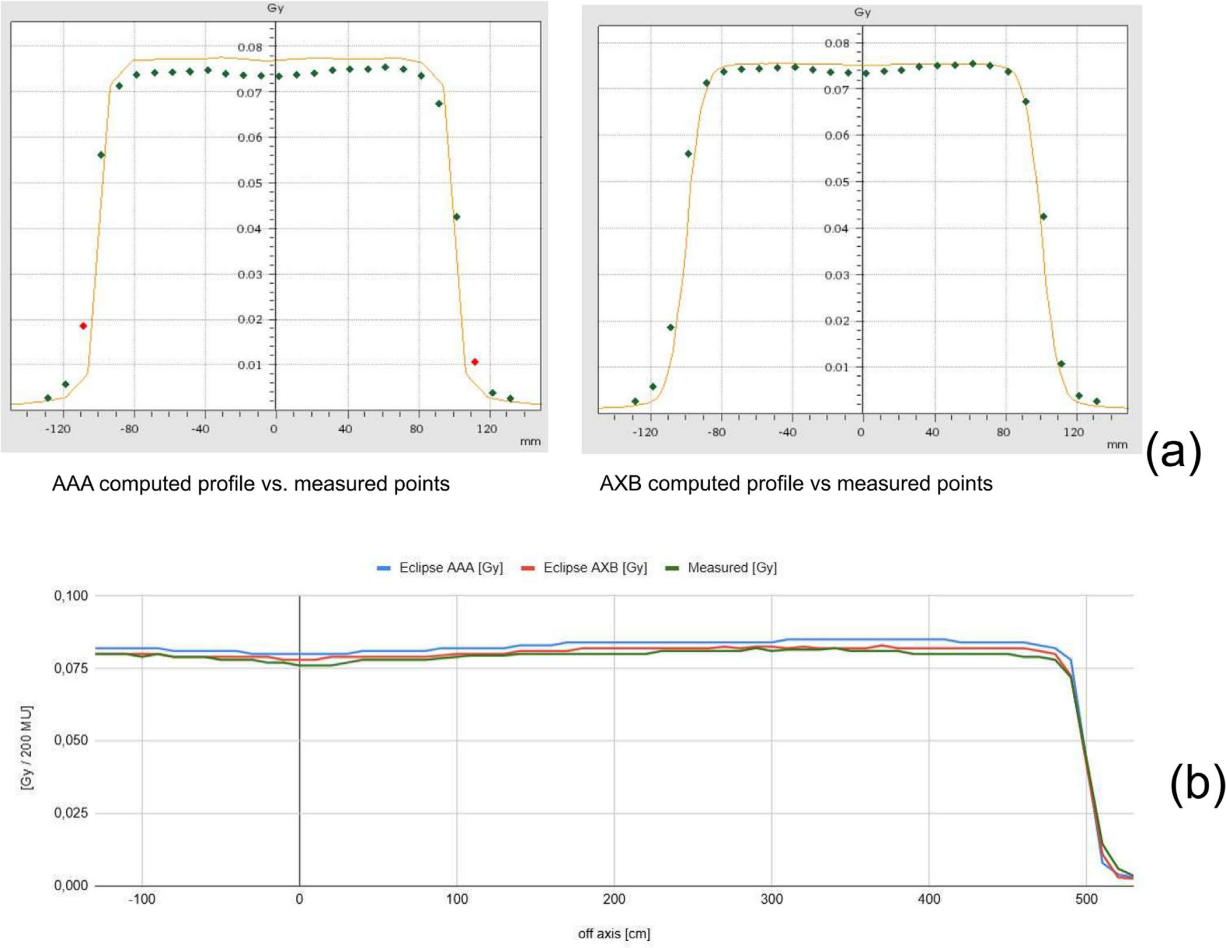
2D array dose profiles of a 15MV beam measured with build‐up plate at eSAD 500 cm compared with TPS calculations. (a) for a 4x4 cm^2^ and (b) for a 4x20 cm^2^ treatment fields (20 cm aperture).

Table 2 compiles the commissioning results of the AAA and AXB algorithms at eSAD for each commissioning measurement series (1.a to 1.d) with the metrics: average deviations (AD), standard deviations (SD) between computed and measured doses with associated confidence limits (CL). Figure 8a shows a comparison of measured beam profiles for 4 × 4 cm^2^ fields at eSAD with profiles calculated using AAA and AXB. The AAA calculation exhibits larger deviations from the measurements, of up to approximately 4%, and discretization effects at profile shoulders, whereas AXB closely matches the measured profile in amplitude and remains smooth. Similar deviation is observed for larger field profiles as shown for a 4 × 20 cm^2^ field Figure 8b.

**TABLE 2:** Comparison of AAA and AXB calculations with measurements for each commissioning series at eSAD.

Commissioning measurement series	Measurement definition (Phantoms positioned at eSSD 485 cm with build‐up plate)	Criterion IAEA for CL*	AAA			AXB		
			AD	SD	CL	AD	SD	CL
1.a.	DEPTH DOSE –Open fields 2 × 2 cm^2^, 5 × 5 cm^2^, 10 × 10 cm^2^, 40 × 40 cm^2^ –30 × 30 × 30 cm^3^ water phantom –Positioned on central axis –Measurement depth in water: 1 to 20 cm	≤4% (depth > 3 cm)	2,66%	1,57%	5,00%*	0,57%	1,51%	2,83%
		≤15% (depth ≤ 3 cm)	4,03%	2,68%	8,05%	0,83%	0,96%	2,27%
1.b.	PROFILES –Open field 10 × 40 cm^2^ –Depth in water: 5cm, 10 cm, 15 cm. –30 × 30 × 30 cm^3^ water phantom positioned at 500 cm eSAD. –Measurement every 10 cm lateral from central axis over 90% of field width.	≤3%	2,17%	0,42%	2,80%	1,18%	0,37%	1,74%
1.c	SLIDING WINDOW –Sliding window of 2; 4; 6; 10; 14; 16; 20 mm × 10 cm –30 × 30 × 30 cm^3^ water phantom. –measurements at 5; 10; 15 cm depth	≤4%	0,47%	1,22%	2,29%	−0,69%	1,48%	2,90%
1.d.	INHOMOGENEITIES Open fields 4 × 4 cm^2^, 10 × 10 cm^2^, 10 × 40 cm^2^ & asymmetric fields –CIRS Thorax Phantom at eSAD 500 cm, chamber in left lung insert. Phantom positioned on axis and 70cm off axis.	≤4%	−2,57%	1,58%	4,95%*	1,70%	1,18%	3,47%

AD: Average deviations, SD: standard deviations, CL: confidence limits (CL). CL values failing IAEA criterion are marked with (*).

### 3.2 Define and validate a standardized treatment planning protocol and workflow

**FIGURE 9:**
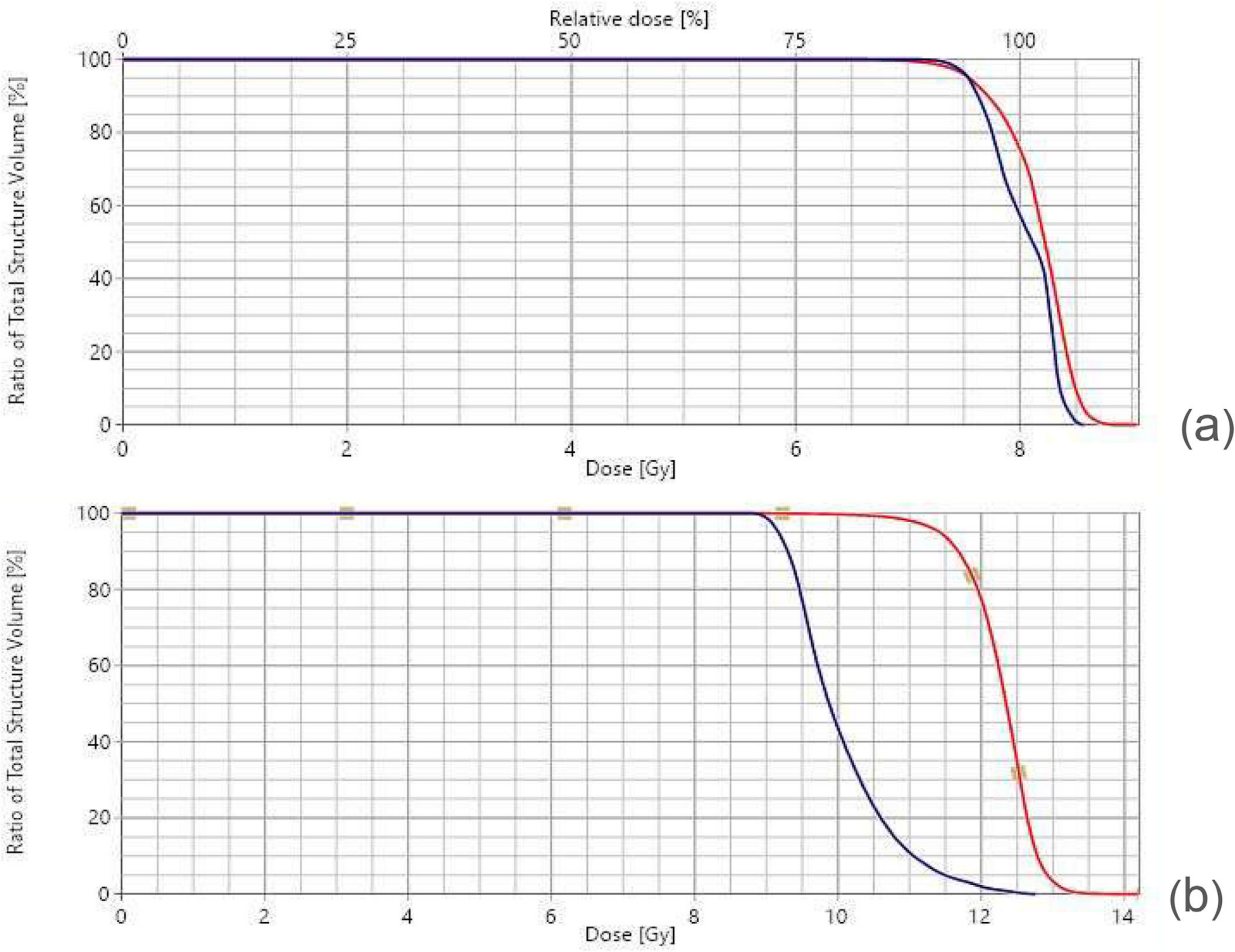
DVH for Patient 1's treatment plan computed with AAA and sliding window fields: red ‐ PTV (body with 3 mm inner margins), blue both lungs (no margin); (a) PD 8 Gy, (b) PD 12 Gy with 2 Gy lung dose reduction.

Treatment planning on three distinct CT datasets demonstrated consistent PTV coverage for all 6 evaluated treatment plans. Representative dose volume histogram (DVH) for Patient 1 is shown in Figure 9, while aggregated DVH metrics for all three patients are shown in Table 3. The total MUs required for the eSAD TBI treatment sub‐plans are shown in Table 4.

**TABLE 3:** DVH metrics from TPS for 12 Gy and 8 Gy plans calculated using AAA algorithm.

		Objectives	Patient 1	Patient 2	Patient 3
			180 cm	160 cm	110 cm
**PD PTV 12 Gy, D Lung 10Gy**	PTV mean	12,00 ± 0,30 Gy	12,28 Gy	12,24 Gy	12,21 Gy
	PTV D98%	≥ 85%	87,0%	86,2%	85%
	PTV V95%	> 90%	95,1%	95,2%	95,3%
	PTV V110%	<5%	2,0%	0,4%	0,5%
	**PTV V120%**	<1%	0,0%	0,0%	0,0%
	**Mean lung D**	< 10 Gy	10,06 Gy	10,02 Gy	9,64 Gy
**PD PV 8 Gy treatment plan**	**PTV Mean**	8,00 ± 0,2 Gy	8,16 Gy	8,12 Gy	8,14 Gy
	**PTV D98%**	≥ 85%	85%	85%	85%
	**PTV V95%**	> 90%	94%	95%	96,6%
	**PTV V110%**	<5%	0,1%	0,1%	0,0%
	**PTV V120%**	<1%	0,0%	0,0%	0,0%

**TABLE 4:** MUs and treatment times for 12 Gy bilateral eSAD fields for three patients, techniques compared.

	Patient 1, 180 cm		Patient 2, 160 cm		Patient 3, 110 cm	
	MUs	irradiation time [min:ss]	MUs	irradiation time [min:ss]	MUs	irradiation time [min:ss]
**12 Gy Solid compensator**						
eSAD patient right	4132	6:53	3756	5:46	3236	5:23
eSAD patient left	4448	7:25	3844	6:24	3144	5:14
**12 Gy, Sliding window**						
eSAD patient right	4519	9:02	4927	9:51	4098	8:12
eSAD patient left	4716	9:26	4760	9:31	4224	8:27
1**2 Gy, Step & shoot**						
eSAD patient right	3702	07:24	3537	7:04	3321	6:38
eSAD patient left	3814	07:38	3473	6:57	3371	6:44

### 3.3 End‐to‐end test with an anthropomorphic phantom

The treatment planning process conducted in accordance with the SOP, involved approximately two hours of work by an experienced medical physicist. Patient specific QA measurement of the eSAD treatment fields applied to the RW3 phantom is reported in Table 5 and shows good agreement for the central axis as well as for the lung position, 40 cm off axis. Fluences measured with the EPID at SAD 100 cm showed that 99% of points passed the gamma criterion for all eSAD IMRT fields.

**TABLE 5:** QA verification measurements of the end‐to‐end test, sliding window, eSAD treatment sub‐plans applied to a RW3 phantom.

	MUs	Measured [mGy]	AAA [mGy]	AXB [mGy	Dev. AAA to measured	Dev. AXB to measured
On Axis, RW3 phantom cantered at 485 cm SSD, measurement in 6,5 cm depth with build‐up plate						
eSAD patient left	3640	1195	1242	1227	3,90%	2,64%
eSAD patient right	3590	1211	1261	1245	4,04%	2,76%
Sum eSAD	7230	2407	2503	2472	3,97%	2,70%
Off Axis, RW3 phantom at 485 cm SSD (40 cm off axis for left fields −40 cm for right fields), measurement in 6,5 cm depth with build‐up plate						
eSAD patient left	3640	768	785	772	2,18%	0,49%
eSAD patient right	3590	772	789	774	2,23%	0,22%
Sum eSAD	7230	1540	1574	1546	2,20%	0,35%

**FIGURE 10:**
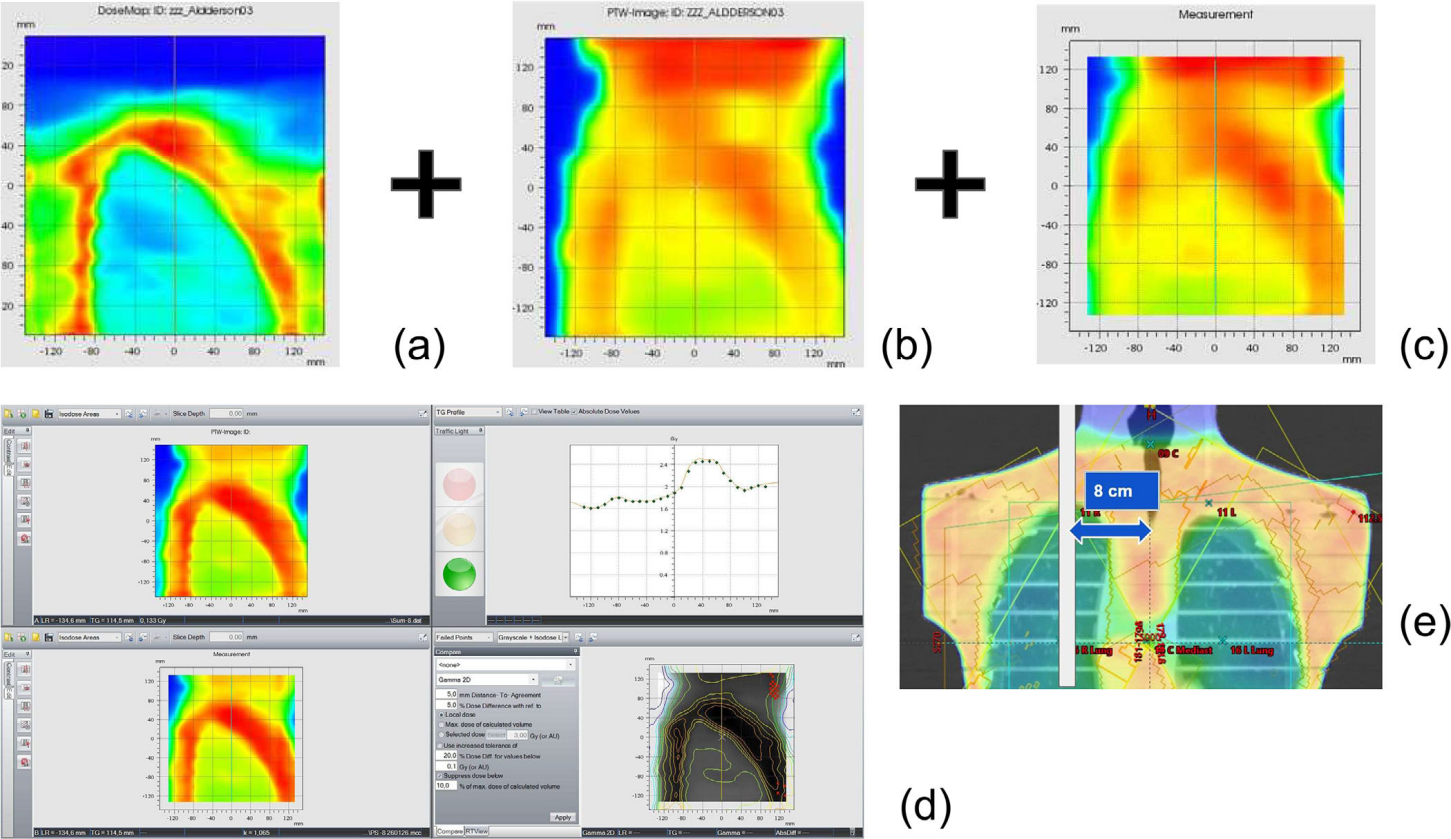
2D array dose profiles measured at 8 cm transverse off‐axis (mid‐right lung position) for the caudal thoracic field junction: (a) SAD VMAT thorax 2D dose profile; (b) eSAD patient right 2D dose profile, array positioned at eSAD 492 cm; (c) eSAD patient left 2D dose profile, array positioned at eSAD 508 cm; (d) Summed dose evaluated in Verisoft; and (e) lateral 2D array position relative to right lung.

**FIGURE 11:**
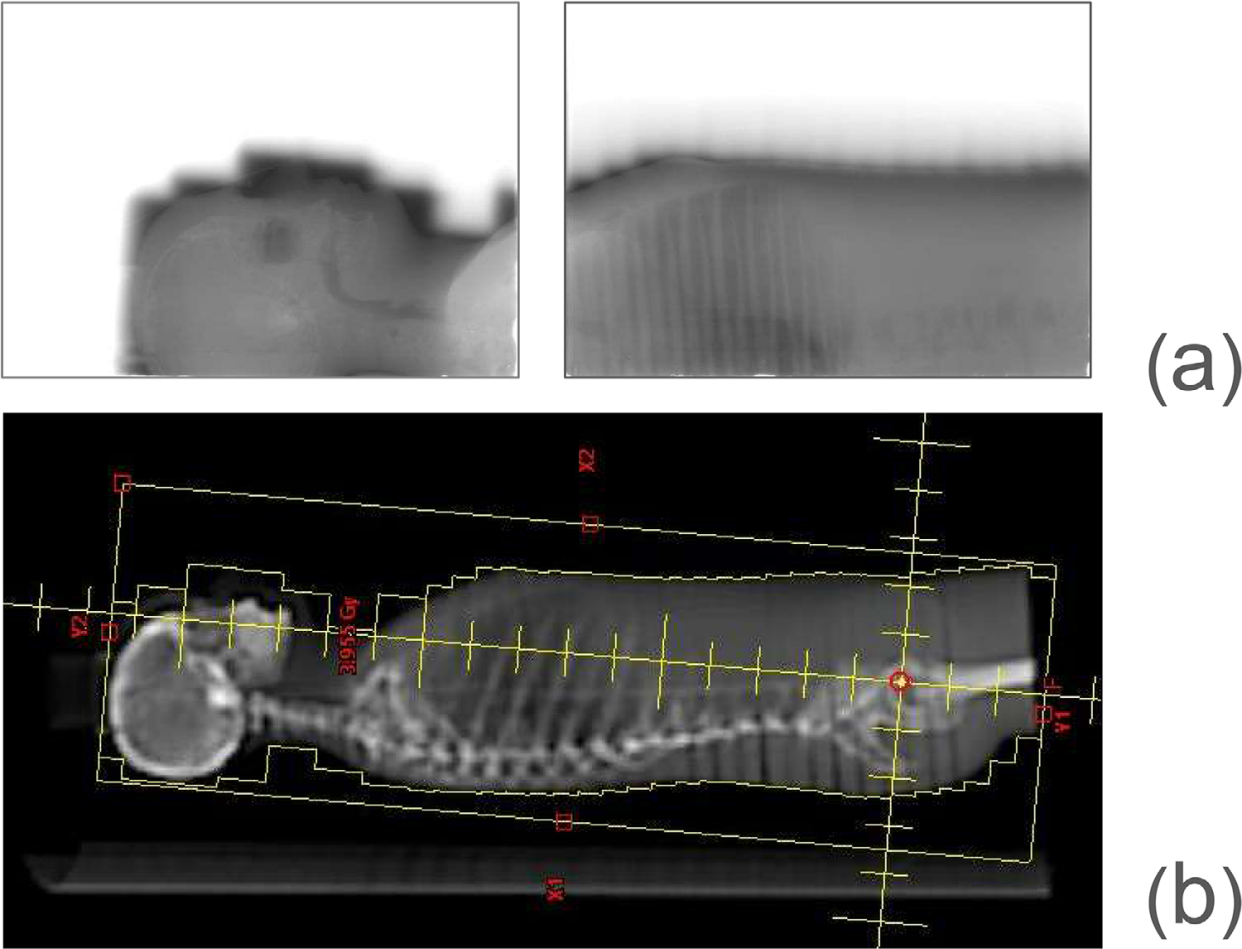
Images obtained with FCR cassettes placed behind the Alderson phantom at eSAD. (b) In comparison, digitally reconstructed radiology setup field conform to the Alderson phantom body contour in the TPS.

The 2D array measurements performed at eSAD verified the accuracy of the dose distribution under eSAD conditions (Table 6), with at least 95% of evaluated points meeting the increased gamma acceptance criteria for the summed dose at the upper and lower field junctions (Figure 10). Radiographic verification provided sufficient anatomical contrast for phantom positioning (Figure 11). Ion chamber measurements for both sliding window and step‐and‐shoot IMRT plans, as summarized in Table 7, showed satisfactory concordance with calculated doses using the AAA and AXB algorithms. For abdominal, lung and head treatment sites, dose deviations remained below 5% for each treatment sub plan delivered as well as for the total fraction dose. Similarly, TLD measurements showed good agreement across all evaluated points (Table 8). The total time to deliver the fraction in the end‐to‐end test was 46 min including position verification of the phantom (Table 9).

**TABLE 6:** End‐to‐end test, gamma analysis results. 2D array profiles vs. AXB dose maps.

Treatment sub‐plan	Coordinates of the 2D dosimetry chamber array centre position [cm]			Points with gamma ≤1 for 5 mm and 5% dose criterion in % (over 1405 points)
	X	Y	Z	
1. Mediastinum cranial				
SAD VMAT thorax	0	0	50	93,9%
eSAD patient right	−500	0	50	90,2%
eSAD patient left*	−500	0	−50	92,6%
**PLAN SUM** *		**0**	**50**	**95,5%**
2. Mediastinum caudal				
SAD VMAT thorax	0	0	30	99,4%
eSAD patient right	−500	0	30	87,8%
eSAD patient left*	−500	0	−30	88,5%
**PLAN SUM** *		**0**	**30**	**99,0%**
3. Right lung cranial				
SAD VMAT thorax	0	0	50	82,7%
eSAD patient right	−492	0	50	95,2%
eSAD patient left*	−508	0	−50	91,0%
**PLAN SUM** *		**0**	**50**	**98,6%**

* The eSAD L IMRT dose profiles were mirrored to be added to SAD VMAT Thorax and eSAD R IMRT profiles to build the plan sum.

**TABLE 7:** Alderson phantom irradiation with SAD VMAT and eSAD IMRT using sliding window MLC motion. Calculated vs. measured values compared.

	Measured [mGy]	AAA [mGy]	AXB [mGy]	AAA deviation to measured	AXB deviation to measured
**Head**					
SAD VMAT thorax	‐*	‐	‐	‐	‐
eSAD patient right	941	972	956	3,32%	1,62%
eSAD patient left	946	988	966	4,40%	2,08%
**Sum**	**1887**	**1960**	**1922**	**3,86%**	**1,85%**
**Right lung**					
SAD VMAT thorax	219**	232	222	6,14%	1,56%
eSAD patient right	826	833	832	0,86%	0,74%
eSAD patient left	527	531	218	0,68%	−1,78%
**Sum**	**1353**	**1364**	**1350**	**0,79%**	**−1,04%**
**Abdomen**					
SAD VMAT thorax	‐*	‐	‐	‐	‐
eSAD Patient right	945	984	968	4,18%	2,48%
eSAD Patient left	908	939	930	3,45%	2,46%
**Sum**	**1852**	**1923**	**1898**	**3,82%**	**2,47%**

^*^Negligible as measurement points not in the direct beam. ** Approximative measurement: ionization chamber placed vertically and not transversal to beam at all gantry angles

**TABLE 8:** AAA/AXB vs. TLD measurements at 14 points in Alderson phantom for one treatment fraction in the end‐to‐end test.

TLD Position (ref. Fig 7)	Measured value [Gy]	AAA [Gy]	AXB [Gy]	AAA deviation to measured	AXB deviation to measured
4	1,86	1,86	1,94	4,14%	−1,71%
5	1,98	1,98	2,06	3,98%	3,04%
9	2,07	2,07	2,05	−0,81%	−0,27%
11 L	1,75	1,75	1,72	−1,76%	−2,01%
11 R	1,62	1,62	1,63	0,37%	−3,94%
16	1,87	1,87	1,80	−3,36%	−5,68%
16 L	1,58	1,58	1,59	0,43%	−3,34%
25	1,96	1,96	2,00	1,83%	−0,26%
25 L	1,97	1,97	2,01	2,14%	0,00%
26	2,07	2,07	1,99	−3,98%	−3,41%
26 R	1,99	1,99	2,02	1,61%	2,18%
32	1,87	1,87	1,95	4,33%	2,68%
32 L	1,97	1,97	2,00	1,57%	0,26%
32 R	2,01	2,01	1,98	4,14%	−1,71%

**TABLE 9:** Duration of a treatment fraction during the end‐to‐end test.

Treatment sub‐plans	Task	Time in min
SAD VMAT thorax	Set‐up & position verification	7
	Irradiation, 4 arcs	5
eSAD patient right	Set‐up & position verification	12
	Irradiation, two fields	7
eSAD patient left	Set‐up & position verification*	8
	Irradiation, two fields	7
**Fraction delivery**		**46**

* TBI couch already at correct vertical position, due to right side eSAD fields

Robustness simulation showed values within or very close to the acceptance criteria for all four simulated shifts (Table 10). Local hotspots, up to PD 120% for one of the position shifts, were observed at the thoracic edges, located at the dose gradient regions of both the thoracic and eSAD IMRT treatment sub‐plans, but, as expected, not in the lungs.

**TABLE 10:** Robustness evaluated by simulating 4 translational shifts of the Alderson phantom for irradiation of the eSAD treatment sub‐plans.

Shift, longitudinal axis	Shift, sagittal axis	PTV V90%	PTV V110%	PTV V120%
		expected >95% PD	expected <5% PD	expected <1% PD
0 cm	0 cm	97,5%	0,3%	0,0%
1 cm	1 cm	98,0%	1,0%	0,0%
1 cm	−1 cm	94,8%	0,2%	0,0%
−1 cm	1 cm	97,3%	2,0%	0,1%
−1 cm	−1 cm	94,9%	0,4%	0,0%

## 4 DISCUSSION

### 4.1 Commissioning of TPS's algorithms at eSAD

Previous studies have evaluated treatment algorithm performance at eSAD, including depth‐dose and profile measurements.^24^, ^26^, ^27^ In 2016, Lamichhane et al.^24^, ^26^ recommended caution when evaluating AAA and AXB (V 11) at eSAD due to accuracy concerns. However, more recent work by Nelson et al.,^24^, ^26^ in 2024 demonstrated that AXB (V 16) provides accurate dose calculations at an eSAD of 500 cm, supporting its use for TBI treatment planning. Our results confirm and complete these last findings through additional measurements specific to our TBI protocol, which includes the use of the build‐up plate and MLC modulation. Deviation to measurements met the IAEA's criteria—defined however for a SAD 100 cm—for AXB but were slightly above for AAA.

Statistical CL depend on how representative the measurement points are. Thus, they should be seen as indicative, not absolute, and useful for identifying calculation model limitations, for example, AAA inaccuracies at shallow depths with a build‐up plate as shown in the PDD measurements results Table 2. Profile measurements obtained with the 2D array were limited by its spatial resolution, however, they still indicate the superior performance of AXB at extended distances. Overall, AAA v16 and AXB v16 are acceptable for planning and optimizing TBI treatment at eSAD at our institution. AXB demonstrates superior accuracy, therefore is more appropriate for final dose calculation and for dose reporting in organs at risk. Their successful commissioning supports clinical implementation and future development of extended‐distance treatment approaches.

### 4.2 Define and validate a standardized treatment planning protocol and workflow

Improved PTV dose conformity was achieved in the PD 8 Gy treatment plans using exclusively the eSAD setup. In contrast, the PD 12 Gy treatment plans, planned with the hybrid method showed localized over‐ or under dosages in the field superpositions area at the cranial and caudal edges of the thoracic volume. Consistent results across all three patient anatomies, with closely matching DVHs, support the reliability of our standardized planning approach. In this study, a lung dose reduction by 10 Gy was set as the objective; however, since this reduction primarily depends on the VMAT treatment sub‐plan, even stronger dose reductions are theoretically possible, considering that a mean lung dose of 8 Gy is a proposed objective in recent protocols.^7^ Additionally, the VMAT treatment sub‐plan could include further VMAT fields using an isocenter positioned below the thorax to reduce the renal dose. This makes the proposed method a potentially simpler alternative to the widely studied multi‐isocenter VMAT TBI techniques, which often require complex planning steps and automated scripting.^8^, ^9^, ^10^, ^28^ Recommendations for dose prescription, delineation definitions and reporting protocols are currently suggested^5^ but global consensus and standards are strongly needed to enable objective comparison of techniques.^7^

The step‐and‐shoot lateral fields required approximately 3700 MUs per patient side, in a similar range as those used with solid compensation (typically 3500 to 4300 MU). In solid compensation, extra MUs account for beam attenuation in the compensator, while in the virtual compensator approach, extra MUs are used for fluence modulation. Sliding window IMRT slightly improved PTV dose homogeneity but increased total MUs by about 15%–20%. The estimated treatment time for eSAD fields remained below 10 min per patient side, which is clinically acceptable and consistent with institutional experience. Additional patient setup on the radiotherapy couch at SAD 100 cm requires approximately 15–20 minutes, based on the typical setup times observed for standard thoracic radiotherapy treatments.

### 4.3 End‐to‐end test using an anthropomorphic phantom

Although an end‐to‐end test does not constitute full validation, as it represents only a single case, it nevertheless confirmed the practical feasibility of the novel hybrid method and reinforced confidence in the field‐junction management approach.

The quality assurance procedure foreseen prior to patient treatment provides a reliable assessment of the delivered dose. EPID fluence measurements at 100 cm provide only relative verification of each treatment field at an eSAD of 500 cm; this limitation is complemented by absolute point‐dose measurements performed in the RW3 phantom.

Fluence measurements performed with the 2D array demonstrated that the adopted treatment planning strategy resulted in a robust summed dose distribution. Although a more comprehensive 3D analysis, such as that performed by Solis et al.^29^ could provide additional insight, the present results, combined with TPS simulations of positional shifts, already indicate that the strategy effectively mitigates the impact of positional uncertainties on the dose distribution. In clinical practice, positioning errors, which are typically greater with patients than with phantoms, must be minimized through effective patient immobilization, with the residual impact expected to be reduced by interfractional variations. Ion chamber measurements showed that both step‐and‐shoot and sliding window are practicable. Although TLD measurements showed somewhat larger deviations—attributable to their higher intrinsic uncertainty^30^—they remained within acceptable limits, with deviations below 5% relative to both AAA and AXB calculations.

## 5 CONCLUSION

The commissioning of the TPS AAA and AXB algorithms for eSAD applications, including MLC modulation, has established the feasibility of TPS‐based planning for TBI at extended distances for our institution. AXB demonstrated superior accuracy to AAA, with CL of 3.5% for evaluated points versus 5% for AAA, and is therefore recommended for final dose calculation.

It is shown that VMAT plans at SAD 100 cm can be integrated with eSAD 500 cm treatment sub‐plans in the TPS, effectively combining the lung‐sparing advantages of VMAT with the geometric robustness of large field irradiation. This hybrid technique offers a technically advanced and clinically viable alternative to existing TBI approaches, and yields a major benefit from the advantage of a commercial TPS for plan evaluation and dose reporting.

## AUTHOR CONTRIBUTIONS

**Marc Delaperrière**: Study design, methodology, data collection and measurements, data analysis and interpretation, drafting and editing of the manuscript. **Christian Felix Schulz**: Contribution of medical expertise and manuscript review. **Markus Hirt**: Methodological contribution and manuscript review. **Frank André Siebert**: Supervision, intermediate and final manuscript reviews.

## CONFLICT OF INTEREST STATEMENT

The authors declare no conflicts of interest regarding this manuscript.

## ETHICS STATEMENT

Treatment planning utilized anonymized CT datasets acquired under institutional ethics committee approval (AZ D 450/24) and with general consent from patients or their legal guardians.

## AI USE DISCLOSURE

AI was used solely for language polishing. All scientific content and conclusions are the authors’ own.
